# Influencing factors of type 2 diabetes remission: a systematic review and meta-analysis

**DOI:** 10.3389/fendo.2026.1916734

**Published:** 2026-10-15

**Authors:** Siwen Cheng, Jinying Chen, Xinlu Zhang, Haotian Qi, Xiaoxia Kang, Weichen Nie, Yilin Feng, Rui Chen

**Affiliations:** 1 Changchun University of Chinese Medicine, Changchun, China; 2 Afliated Hospital of Changchun University of Chinese Medicine, Changchun, China; 3 Department of Tuina, Affiliated Hospital of Changchun University of Chinese Medicine, Changchun, China

**Keywords:** influencing factors, meta-analysis, systematic review, T2DM, type 2 diabetes remission

## Abstract

**Purpose:**

To identify factors influencing type 2 diabetes mellitus (T2DM) remission and compare differences across intervention types and remission criteria.

**Methods:**

T2DM remission was defined as HbA1c < 6.5% (48 mmol/mol), measured at least 3 months after cessation of glucose-lowering pharmacotherapy. Relevant literature on factors influencing T2DM remission was searched in English-language databases, with the search updated through March 2026. Meta-analysis was performed using Review Manager 5.4 and Stata 18.0 software.

**Results:**

A total of 36 studies were included. Higher fasting plasma glucose (OR = 0.99, 95% CI = [0.99, 1.00], P < 0.001), higher glycated hemoglobin (OR = 0.91, 95% CI = [0.88, 0.94], P < 0.001), longer disease duration (OR = 0.89, 95% CI = [0.88, 0.90], P < 0.001), and a greater number of oral antidiabetic agents (OR = 0.56, 95% CI = [0.38, 0.81], P = 0.002) were associated with lower odds of remission. A greater percentage of total weight loss was associated with higher odds of remission (OR = 1.05, 95% CI = [1.04, 1.07], P < 0.001). In surgical interventions, a higher body mass index (OR = 1.05, 95% CI = [1.02, 1.08], P = 0.002) was associated with higher odds of remission, whereas older age (OR = 0.96, 95% CI = [0.94, 0.98], P < 0.001) and insulin use (OR = 0.34, 95% CI = [0.23, 0.52], P < 0.001) were associated with lower odds of remission. In non-surgical interventions, alcohol cessation was associated with higher odds of remission (OR = 1.14, 95% CI = [1.05, 1.26], P < 0.001), while factors influencing lower odds of remission included smoking (OR = 0.90, 95% CI = [0.86, 0.94], P < 0.001), continued alcohol consumption (OR = 0.90, 95% CI = [0.84, 0.95], P < 0.001), and a higher body mass index (OR = 0.89, 95% CI = [0.86, 0.92], P < 0.001). Gender, race/ethnicity, physical exercise, intermittent fasting, HOMA-IR and C-peptide level were not associated with T2DM remission.

**Conclusion:**

T2DM remission is influenced by multiple factors, with BMI showing opposite associations by intervention type. Future large-scale, prospective studies are needed to validate these findings.

**Systematic review registration:**

https://www.crd.york.ac.uk/PROSPERO/, identifier CRD420261295582.

## 1 Introduction

As a prevalent chronic metabolic disorder, type 2 diabetes mellitus (T2DM) continues to increase in incidence across populations worldwide (1). Traditionally, T2DM has been viewed as an irreversible condition requiring patients to take antidiabetic medications for life to control blood glucose levels. However, a large body of studies has shown that through active intervention, some T2DM patients can maintain normal blood glucose levels even after discontinuing antidiabetic medications (2, 3). In 2021, the American Diabetes Association (ADA) released a consensus report, defining type 2 diabetes remission uniformly as: HbA1c <6.5% (48 mmol/mol), measured at least 3 months after cessation of glucose lowering pharmacotherapy (4).

Previous studies have reported factors potentially associated with remission of T2DM, including age, disease duration, body mass index (BMI), HbA1c, and percentage of total weight loss (%TWL) (5–7). However, findings across these investigations remain inconsistent, and the definition of “remission” is not standardized. Existing systematic reviews and meta-analyses either focus on a single influencing factor or a specific intervention modality (8, 9), or solely address treatment efficacy (10). No study has yet systematically evaluated the differential effects of multiple factors between surgical and non-surgical interventions from the perspective of influencing factors. Surgical and non-surgical interventions differ fundamentally in terms of weight loss magnitude and metabolic mechanisms. Surgical interventions primarily regulate the body’s metabolism, appetite, and energy balance by altering the anatomical structure of the gastrointestinal tract, thereby inducing significant changes in the secretion of gut hormones (11). Non-surgical interventions, on the other hand, primarily work by reducing ectopic fat deposits in the liver and pancreas, thereby restoring hepatic insulin sensitivity and correcting β-cell dysfunction (12). Since the two approaches act through different mechanisms, the same factor may have varying effects on T2DM remission under different intervention modalities. Therefore, we used intervention type as the core subgroup variable to investigate whether the associations between various influencing factors and T2DM remission differ across intervention mechanisms, thereby providing evidence to inform individualized treatment strategies under different intervention modalities.

The purpose of this study is to systematically evaluate factors influencing T2DM remission in all patients with type 2 diabetes who underwent surgical (bariatric/metabolic surgery) or non-surgical (lifestyle interventions or pharmacotherapy) interventions. Specifically, we aimed to: (1) identify factors associated with T2DM remission, including gender, age, race/ethnicity, lifestyle factors, BMI, fasting plasma glucose (FPG), HbA1c, HOMA-IR, C-peptide levels, disease duration, number of oral antidiabetic agents, insulin use, and %TWL; (2) compare differences in these factors between surgical and non-surgical interventions; and (3) provide evidence to guide clinicians in developing individualized treatment strategies for T2DM remission.

## 2 Materials and methods

### 2.1 Search strategy

Relevant literature on the factors influencing remission of T2DM was searched in PubMed, Web of Science, Embase, and the Cochrane Library, with articles published up to March 20, 2026 included. Clinical trial registries and grey literature were not searched. Search terms included Diabetes Mellitus, Type 2; T2DM; remission; resolution; risk factor; and prognosis (**Supplementary Table 1**). This study was prospectively registered on the PROSPERO platform on January 28, 2026 (CRD420261295582), before the literature screening and data extraction. All reported analyses were prespecified.

### 2.2 Inclusion criteria

1. Population (P): All patients with T2DM; 2. Intervention (I): Surgical interventions (bariatric/metabolic surgery, including Roux-en-Y gastric bypass, sleeve gastrectomy, and other bariatric procedures) or non-surgical interventions (lifestyle interventions and pharmacotherapy); 3. Exposure (E): Various factors potentially influencing T2DM remission, including gender, age, race/ethnicity, lifestyle factors, BMI, FPG, HbA1c, HOMA-IR, C-peptide levels, disease duration, number of oral antidiabetic agents, insulin use, and %TWL, among others; 4. Outcome (O): T2DM remission with a follow-up period of ≥ 6 months; 5. Study design (S): Prospective or retrospective cohort studies explicitly reporting predictors, influencing factors, or risk factors for T2DM remission, with multivariate unconditional logistic regression as the primary statistical method and providing combinable statistical data.

### 2.3 Exclusion criteria

1. For multiple publications from the same research team, only the most comprehensive and most recently published study will be included; 2. Studies lacking sufficient outcome data or data sufficient for analysis; 3. Publications for which the full text is unavailable.

### 2.4 Study selection and data extraction

Two researchers independently searched for, screened, and extracted data from relevant literature, cross-checking their findings with one another. In the event of a disagreement during this process, a third researcher made the final decision. The extracted information included: first author, publication date, study region, study type, sample size, baseline disease duration, baseline BMI, baseline age, baseline HbA1c, gender, and influencing factors included in the logistic regression analysis.

### 2.5 Quality assessment

Quality assessment of the cohort studies was conducted by applying the Newcastle-Ottawa Scale (NOS). The overall quality rating spans from 0 to 9, where studies scoring between 0 and 5 are classified as low quality, those receiving 6 or 7 points are deemed moderate quality, and those achieving 8 or 9 points are designated as high quality.

### 2.6 Statistical analysis

Meta-analyses were conducted using Review Manager 5.4 and Stata 18.0 software. Effect measures were analyzed by calculating pooled odds ratios (ORs) and 95% confidence intervals (CIs). For continuous predictors, odds ratios are reported per the following unit increments: age per 1 year; BMI per 1 kg/m²; fasting plasma glucose per 1 mg/dL; HbA1c per 1%; HOMA-IR per 1 unit; C-peptide per 1 ng/mL; diabetes duration per 1 year; number of oral antidiabetic agents per 1 agent; total weight loss per 1%. When original studies reported continuous predictors in different units, effect estimates were converted to the reference unit using standard conversion factors (e.g., HbA1c: 1% = 10.93 mmol/mol; fasting plasma glucose: 1 mmol/L = 18 mg/dL; C-peptide: 1 ng/mL = 0.331 pmol/L). For log-transformed effect estimates, the OR was rescaled using the formula: OR _standardized_ = exp[ln(OR _original_) × (unit _standardized/_unit _original_)]. Studies for which unit conversion was not possible were excluded from the pooled analysis for that predictor. Heterogeneity tests on the included studies guided model selection: I² < 50% or P ≥ 0.05 called for a fixed-effects model, otherwise, a random-effects model was applied. Whenever a particular study could not be incorporated into the meta-analysis, a descriptive evaluation method was used instead. To examine heterogeneity, both subgroup and sensitivity analyses were applied. Subgroup analysis was performed only when at least five studies were available for inclusion and each subgroup contained no fewer than two studies. Sensitivity analyses were performed using a leave-one-out approach and by comparing fixed-effects and random-effects models. Egger’s test was used to assess publication bias for factors with ≥ 10 included studies, and funnel plots were constructed. A P-value < 0.05 was considered statistically significant in all analyses.

### 2.7 Subgroup definitions

Subgroup analyses were performed according to intervention type and T2DM remission criteria. For the intervention type subgroup, studies were stratified by intervention modality into surgical and non-surgical. Surgical interventions were defined as bariatric/metabolic surgical procedures, including Roux-en-Y gastric bypass, sleeve gastrectomy, and mixed bariatric/metabolic procedures without distinction by specific technique. Non-surgical interventions encompassed all other treatment modalities, including lifestyle interventions (dietary modification, physical exercise) and pharmacological therapy. Where sufficient data were available, further subgroup analyses were conducted by specific surgical procedure (Roux-en-Y gastric bypass, sleeve gastrectomy, or mixed procedures). For the remission criteria subgroup, studies were stratified by the stringency of the T2DM remission definition into strict remission and non-strict remission. In accordance with the 2021 ADA Expert Consensus Report, this study defined “strict remission criteria” as meeting the following two core conditions: 1) complete discontinuation of all antidiabetic medications for at least 3 months; 2) glycated hemoglobin (HbA1c) remaining < 6.5% after discontinuation (4). Any remission outcome that simultaneously met the above two criteria (including partial remission) was classified as strict remission. Non-strict remission criteria referred to other remission definitions that did not fully satisfy these two conditions, including but not limited to: glycemic control achieved with reduced rather than complete withdrawal of glucose-lowering medications, no specified duration of medication discontinuation, or the inclusion of endpoints such as “glycemic improvement” in the remission definition.

## 3 Results

### 3.1 Literature retrieval and screening

A total of 15,980 articles were retrieved from PubMed (n=3,986), Embase (n=6,016), Web of Science (n=5,099), and the Cochrane Library (n=879), and no grey literature was manually retrieved from reference lists. After removing 5,639 duplicate records and excluding 10,175 articles following a review of titles and abstracts, 36 articles were ultimately included following full-text screening (**Figure 1**) (7–9, 13–45).

**Figure 1:**
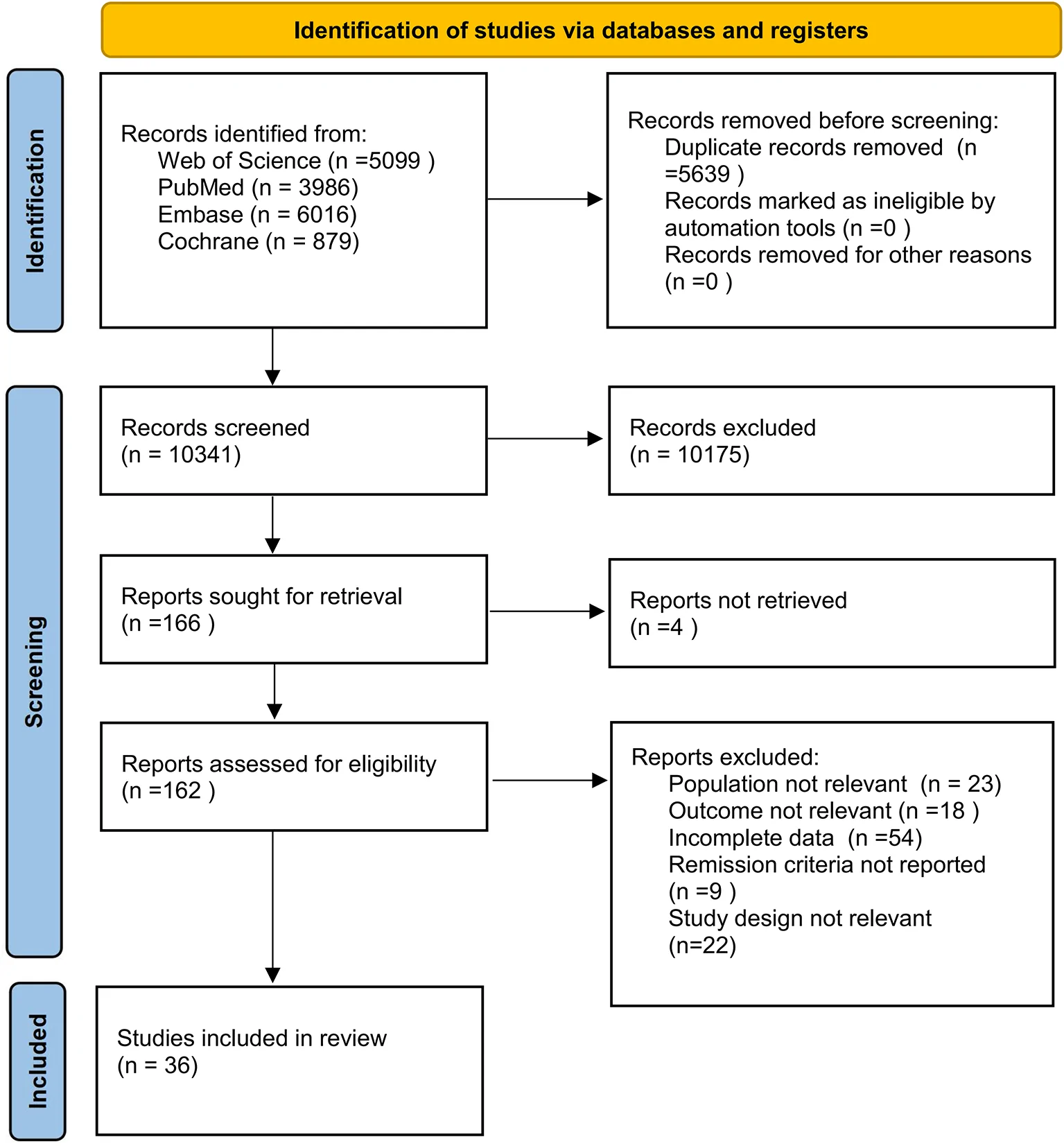
PRISMA diagram showing selection of articles for review.

### 3.2 Study characteristics and quality assessment

A total of 36 studies were included (5–7, 13–45), comprising 30 retrospective cohort studies (5–7, 13–23, 25, 28–32, 34, 35, 37, 38, 40–45) and six prospective cohort studies (24, 26, 27, 33, 36, 39). The total sample size of this study was 784,710 cases (**Table 1**; **Supplementary Table 2**). All 36 studies were published between 2016 and 2026, with 29 studies (5, 7, 13–15, 19, 21–26, 28–34, 36–45) published after 2021 and the newest coming out in 2026 (24). This temporal distribution suggests the topic is very current, which provides a strong justification for conducting a meta-analysis. This study pooled studies that mentioned the same risk factors in ≥ 2 studies. The quality assessment showed that among the 36 included studies, 10 studies (15–17, 20, 26, 29, 30, 35, 42, 45) were of moderate quality and 26 studies (5–7, 13, 14, 18, 19, 21–25, 27, 28, 31–34, 36–41, 43, 44) were of high quality (**Supplementary Table 3**).

**Table 1:** Characteristics of the studies included in the meta-analysis.

First author	Country		Sample size	Follow-up (months)	Study design	Disease duration (years)	BMI (kg/m2)	Age (years)	HbA1c (%)		Female (%)
Agha A (13)	UAE		238	60	Retrospective	8.30 ± 4.20	41.40 ± 6.70	46.80 ± 11.60	7.80 ± 1.50		67.6
Ahmadi MA (14)	Pakistan		81	12	Retrospective	8.30 ± 3.60	42.60 ± 5.20	47.20 ± 8.50	8.70 ± 1.20		NR
Allum M (15)	UAE		541	12	Retrospective	NR	40.60 ± 5.70	41.60 ± 10.90	7.20 ± 1.40		66.0
Aminian A (16)	USA		134	72	Retrospective	7.70 ± 6.00	46.90 ± 12.10	52.30 ± 10.40	8.20 ± 1.80		NR
Chen M (5)	USA		9900	12	Retrospective	NR	34.10 ± 8.10	60.50 ± 13.00	8.20 ± 1.90		54.0
Coelho D (17)	Brazil		66	52	Retrospective	8.67 ± 8.73	41.85 ± 11.39	46.05 ± 9.36	8.42 ± 1.67		74.2
Dang JT (18)	Canada		207	12	Retrospective	7.60 ± 6.70	46.30 ± 8.10	49.70 ± 8.80	6.90 ± 1.30		73.9
Dowgiałło-gornowicz N (19)	Poland		146	50	Retrospective	NR	42.92 ± 6.04	66.90 ± 1.52	NR		61.0
Elgenaied I (20)	Qatar		230	12	Retrospective	NR	43.53 ± 6.98	45.66 ± 8.84	8.13 ± 1.75		69.1
Gao X (21)	China		95	72	Retrospective	6.59 ± 1.36	29.78 ± 1.78	49.90 ± 8.11	9.71 ± 2.19		64.2
Gao X (22)	China		34	12	Retrospective	5.94 ± 1.76	30.90 ± 1.75	42.15 ± 7.41	8.65 ± 1.89		55.9
Ghusn W (23)	USA		961	67	Retrospective	9.00 ± 7.80	45.70 ± 8.30	52.80 ± 10.90	7.50 ± 1.50		67.0
Giudicelli G (24)	France		201	60	Prospective	7.53 ± 8.73	45.00 ± 5.90	14.80 ± 10.70	6.90 ± 1.70		72.6
Jans A (6)	Sweden		8546	24	Retrospective	NR	42.20 ± 5.80	47.80 ± 10.10	7.50 ± 3.70		61.7
Kim J (25)	Korea		138211	96	Retrospective	NR	25.69 ± 3.41	54.93 ± 10.61	NR		41.0
Liu H (26)	China		40	6	Prospective	NR	30.57 ± 3.08	39.22 ± 11.69	7.24 ± 0.39		45.0
Luo Y (27)	China		87	48	Prospective	6.40 ± 4.80	31.29 ± 6.51	44.20 ± 11.20	8.01 ± 1.70		69.0
Mateo-Gavira I (28)	Spain		83	24	Retrospective	8.00 ± 9.42	50.44 ± 5.68	44.13 ± 10.38	9.10 ± 2.20		61.4
Matsui T (7)	Japan		1903	60	Retrospective	0	26.30 ± 4.20	48.30 ± 5.30	NR		0
First author	Year	Country	Sample size	Follow-up (months)	Study design	Disease duration (years)	BMI (kg/m2)	Age (years)		HbA1c (%)	Female (%)
Meira I (29)	2024	Portugal	95	120	Retrospective	NR	44.90 ± 6.39	48.79 ± 9.08		7.00 ± 1.48	84.0
Moradi M (30)	2022	Iran	1351	12	Retrospective	5.70 ± 5.90	43.70 ± 6.80	47.30 ± 10.00		7.80 ± 1.80	78.8
Morieri ML (31)	2025	Italy	13277	48	Retrospective	1.00 ± 1.30	30.10 ± 5.70	61.60 ± 12.70		7.40 ± 1.70	41.1
Nguyen NTK (32)	2022	China	119	12	Retrospective	NR	42.20 ± 7.00	41.90 ± 9.50		7.93 ± 1.67	53.7
Niu L (33)	2024	China	95	27	Prospective	6.35 ± 3.76	39.31 ± 7.45	36.76 ± 11.29		8.46 ± 2.56	53.7
Pan Y (34)	2022	China	90	60	Retrospective	NR	42.20 ± 7.00	41.90 ± 9.50		7.93 ± 1.67	53.7
Park JY (35)	2016	Korea	134	12	Retrospective	4.60 ± 5.80	37.90 ± 5.20	42.30 ± 11.1		8.00 ± 1.50	77.6
Salman AA (36)	2021	Egypt	226	24	Prospective	7.40 ± 10.45	43.10 ± 4.60	41.60 ± 8.80		7.70 ± 1.20	43.4
Shen SC (37)	2021	Asia	1016	12	Retrospective	3.90 ± 4.80	39.50 ± 7.00	42.10 ± 10.40		8.10 ± 1.60	55.5
Silva MM (38)	2022	Portugal	403	12	Retrospective	NR	45.00 ± 6.70	47.00 ± 10.10		6.80 ± 1.40	79.4
Stenberg E (39)	2021	Sweden	8057	24	Prospective	3.70 ± 4.59	42.20 ± 5.74	47.40 ± 10.08		7.55 ± 1.58	61.7
Thapa B (40)	2025	USA	556758	36	Retrospective	NR	NR	59.00 ± 13.00		NR	46.3
Tripathi P (41)	2025	India	1897	12	Retrospective	NR	27.28 ± 3.71	NR		7.64 ± 1.48	39.9
Vangoitsenhoven R (42)	2021	USA	519	96	Retrospective	6.53 ± 6.32	45.38 ± 7.66	51.60 ± 9.67		7.50 ± 1.56	67.1
Wu H (43)	2024	China	37326	95	Retrospective	0	26.40 ± 4.02	56.60 ± 9.90		7.70 ± 1.80	49.5
Wu Z (44)	2025	China	1440	12	Retrospective	NR	36.49 ± 6.16	35.46 ± 8.62		7.70 ± 1.93	100
Zhang N (45)	2025	China	203	12	Retrospective	12.65 ± 3.73	32.80 ± 4.90	48.50 ± 8.40		8.80 ± 1.80	60.6

### 3.3 Patient-related factors

#### 3.3.1 Gender

A total of eight studies (5, 6, 21, 28, 30, 38–40) evaluated the association between gender and remission of T2DM. Due to the high heterogeneity of the data (I² = 90%, P < 0.001), a random-effects model was used. The results showed no statistically significant association between gender and T2DM remission (OR = 0.88, 95% CI = [0.73, 1.07], P = 0.20; **Figure 2A**). Subgroup analysis by T2DM remission criteria showed that for studies adopting a strict definition of remission, the pooled OR was 1.10 (95% CI = [0.99, 1.23], I² = 63%, P = 0.080), whereas for studies using a non-strict definition, the pooled OR was 0.79 (95% CI = [0.64, 0.98], I² = 75%, P = 0.04). Subgroup analysis by intervention type showed that the overall pooled OR for the surgical group was 1.01 (95% CI = [0.77, 1.33], I² = 93%, P < 0.001), and for the non-surgical group, the overall pooled OR = 0.82 (95% CI = [0.64, 1.05], I² = 81%, P < 0.001) (**Supplementary Table 4**). Neither subgroup showed a statistically significant association between gender and T2DM remission. Subgroup analysis showed that T2DM remission criteria were a significant source of heterogeneity, with a significant difference between the strict and non-strict definition subgroups (P = 0.007). In contrast, intervention type did not significantly reduce heterogeneity, and the difference between surgical and non-surgical subgroups was not significant (P = 0.280), suggesting that intervention type may not be a major contributor to heterogeneity.

**Figure 2:**
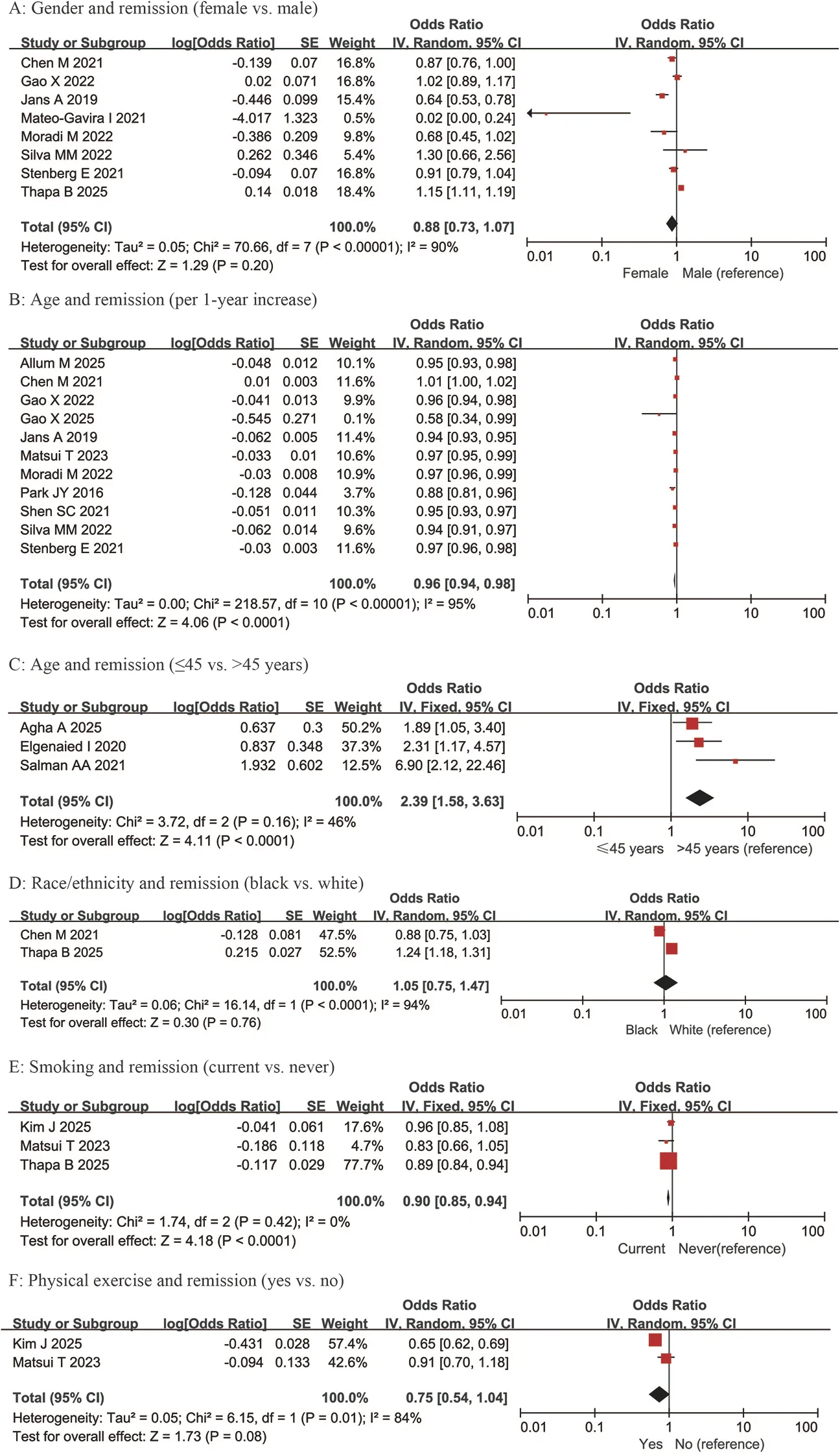
Forest Plot of Patient-Related Factors. **(A)** Gender; **(B)** Age (per 1-year increase); **(C)** Age (≤45 vs. >45 years); **(D)** Race/ethnicity; **(E)** Smoking; **(F)** Physical exercise.

#### 3.3.2 Age

A total of 19 studies (5–7, 13–15, 20–22, 24, 27, 30, 35–41) evaluated the association between age and remission of T2DM. Of these, 11 studies (5–7, 15, 21, 22, 30, 35, 37–39) were included in a meta-analysis of continuous variables, three studies (13, 20, 36) were included in a meta-analysis of categorical variables, and the remaining five studies (14, 24, 27, 40, 41) could not be included in quantitative pooling due to inconsistent reporting and were described only qualitatively. Due to the high heterogeneity (I² = 95%, P < 0.001), a random-effects model was used for the continuous variables. The results suggested that younger age was associated with higher odds of remission (OR = 0.96, 95% CI = [0.94, 0.98], P < 0.001; **Figure 2B**). Subgroup analysis identified T2DM remission criteria as a major source of heterogeneity. Under strict remission definitions, the pooled OR was 0.96 (95% CI = [0.93, 0.98], I² = 44%, P = 0.167); under non-strict remission definitions, the overall pooled OR = 0.96 (95% CI = [0.94, 0.98], I² = 97%, P < 0.001), indicating that this subgroup estimate is less reliable. Between-group differences were significant (P = 0.001). Subgroup analysis by intervention type did not substantially reduce heterogeneity; however, between-group differences were significant (P < 0.001).The pooled OR for the surgical group was 0.95 (95% CI = [0.94, 0.97], I² = 81%, P < 0.001), suggesting a statistically significant association between younger age and higher odds of remission in this subgroup. However, for the non-surgical group, the pooled OR was 0.99 (95% CI = [0.95, 1.03], I² = 94%, P < 0.001), with the confidence interval crossing the null value, indicating no statistically significant association in this subgroup. Due to low heterogeneity, a fixed-effects model was used for the classification variable (I² = 46%, P = 0.15). The results indicated that, compared with patients aged >45 years, those aged ≤45 years had higher remission odds for type 2 diabetes, with an overall pooled OR of 2.39 (95% CI = [1.58, 3.63], P < 0.001; **Figure 2C**). In addition, Giudicelli et al. (24) Tripathi et al. (41), and Luo et al. (27) all reported that younger age was associated with a higher odds of T2DM remission (P = 0.027, P = 0.043, P = 0.012). Thapa et al. (40) reported that younger patients (aged 18–29) had higher probability of remission compared to patients aged 75 (OR = 1.21, 95% CI = [1.06, 1.38], P < 0.05). In contrast, Ahmadi et al. (14) reported that age was not significantly associated with T2DM remission (≥ 50; OR = 1.50, 95% CI = [0.90, 2.60], P = 0.105).

#### 3.3.3 Race/ethnicity

A total of two studies (5, 40) evaluated the association between race/ethnicity and remission of T2DM. Both studies used White individuals as the reference group to compare the impact of race on T2DM remission in Black individuals. Due to high heterogeneity, a random-effects model was used (I² = 94%, P < 0.001). Race/ethnicity showed no statistically significant association with T2DM remission (OR = 1.05, 95% CI = [0.75, 1.47], P = 0.760; **Figure 2D**). Furthermore, Thapa et al. (40) demonstrated that, when remission rates were contrasted against those of white individuals, both Asian patients (OR = 0.72, 95% CI = [0.68, 0.76], P < 0.05) and Native Hawaiian or other Pacific Islander patients (OR = 0.60, 95% CI = [0.51, 0.70], P < 0.05) showed a reduced frequency of remission. In contrast, Hispanic individuals exhibited an elevated chance of remission (OR 1.10, 95% CI = [1.05, 1.16], P < 0.05) relative to their non-Hispanic counterparts.

#### 3.3.4 Lifestyle factors

Three studies (7, 25, 40) examined smoking, one study (25) examined alcohol cessation, two studies (7, 25) examined physical exercise, and one study (41) examined intermittent fasting. A fixed-effects model analysis showed that smoking was associated with lower odds of T2DM remission (OR = 0.90, 95% CI = [0.85, 0.94], I² = 0%, P < 0.001; **Figure 2E**). A random-effects model analysis showed that physical exercise was not significantly associated with T2DM remission (OR = 0.75, 95% CI = [0.54, 1.04], I² = 84%, P = 0.080; **Figure 2F**). Since only one study each was available for alcohol cessation and intermittent fasting, descriptive analyses were conducted. Kim et al. (25) reported that continued alcohol consumption was associated with lower odds of remission (OR = 0.90, 95% CI = [0.84, 0.95], P < 0.001), whereas alcohol cessation was associated with higher odds of remission (OR = 1.14, 95% CI = [1.05, 1.26], P < 0.001). Tripathi et al. (41) divided patients into two groups based on whether they practiced intermittent fasting, the results showed that intermittent fasting was not a factor influencing T2DM remission (OR = 1.25, 95% CI = [0.91, 1.70], P = 0.159).

### 3.4 Metabolic and laboratory parameters

#### 3.4.1 BMI

A total of 14 studies (6, 7, 15, 19, 21, 22, 26, 28, 30, 33, 35, 37–39) evaluated the association between BMI and remission of T2DM. A random-effects model showed that higher BMI was associated with higher odds of remission (OR = 1.04, 95% CI = [1.00, 1.07], I² = 92%, P = 0.050; **Figure 3A**). We conducted subgroup analyses based on intervention type and remission criteria for T2DM. Heterogeneity exploration showed a reduction in heterogeneity within subgroups. These findings suggest that both intervention type and remission criteria may have contributed to the observed heterogeneity. The pooled OR for the surgical group was 1.05 (95% CI = [1.02, 1.08], I²= 88%, P < 0.001), suggesting that higher BMI was associated with higher odds of remission in this subgroup. In contrast, the pooled OR for the non-surgical group was 0.89 (95% CI = [0.86, 0.92]), with low heterogeneity (I²= 0%, P = 0.467), indicating that higher BMI was associated with lower odds of remission in this subgroup. However, this pooled estimate was derived from only two studies; therefore, this finding should be considered exploratory and interpreted with caution. Further subgroup analyses were performed by specific surgical procedure. The pooled OR for the Roux-en-Y gastric bypass subgroup was 1.07 (95% CI = [1.00, 1.14], I² = 91%, P = 0.044). The pooled OR for the mixed procedures subgroup was 1.04 (95% CI = [1.01, 1.08], I² = 79%, P = 0.019). The between-group difference was statistically significant (P < 0.001), suggesting that the association between BMI and remission differed across procedure types. Subgroup analysis based on T2DM remission criteria showed that under the strict remission definition, the pooled OR was 1.07 (95% CI = [1.03, 1.11], I² = 53%, P = 0.095); under non-strict remission definitions, the pooled OR was 1.02 (95% CI = [0.98, 1.07], I² = 93%, P < 0.001), but with substantially higher heterogeneity (I²= 93%, P < 0.001), suggesting that this subgroup estimate is less reliable. Between-group differences were significant (P < 0.001). In addition, three other studies reported on BMI factors. Coelho et al. (17) demonstrated that, compared with a BMI < 35, higher BMI was associated with a higher rate of T2DM remission (P = 0.002). Thapa et al. (40) divided patients into five BMI groups (< 18.5, 18.5–25, 25–30, 30–35, 35–40), all using BMI ≥ 40 as the reference group. The results showed that the odds of T2DM remission increased with higher BMI categories, suggesting that higher baseline BMI may be associated with higher odds of remission in the surgical population. In contrast, Ahmadi et al. (14) reported that BMI was not significantly associated with T2DM remission (≥ 45 vs. < 45; OR = 1.30, 95% CI = [0.80, 2.30], P = 0.213).

**Figure 3:**
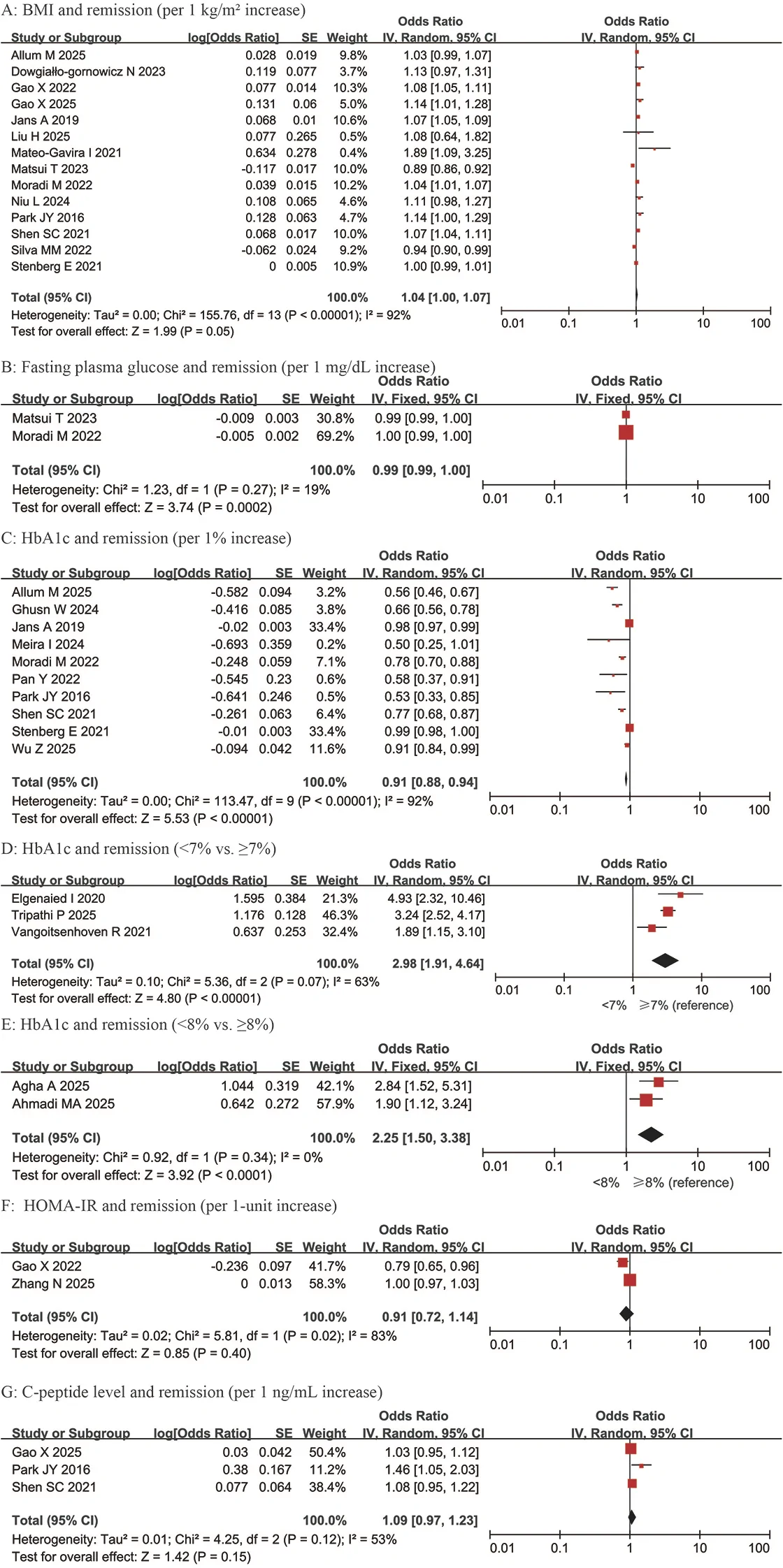
Forest Plot of Metabolic and Laboratory Parameters. **(A)** BMI; **(B)** Fasting plasma glucose; **(C)** HbA1c (per 1% increase); **(D)** HbA1c (<7% vs. ≥7%); **(E)** HbA1c (<8% vs. ≥8%); **(F)** HOMA-IR; **(G)** C-peptide level.

#### 3.4.2 Fasting plasma glucose

A total of two studies (7, 30) were included. Due to low heterogeneity, a fixed-effects model was used (I² = 19%, P = 0.199). A lower fasting plasma glucose was associated with higher odds of remission (OR = 0.99, 95% CI = [0.99, 1.00], I² = 19%, P < 0.001; **Figure 3B**). Additionally, Luo et al. (27) demonstrated that, compared with fasting blood glucose ≥7.0 mmol/L, a lower fasting blood glucose level was associated with higher remission odds (OR = 4.52, 95% CI = [1.11, 18.33], P = 0.026).

#### 3.4.3 HbA1c

A total of 15 studies (6, 13–15, 20, 23, 29, 30, 34, 35, 37, 39, 41, 42, 44) evaluated the association between HbA1c and remission of T2DM. Of these, ten studies (6, 15, 23, 29, 30, 34, 35, 37, 39, 44) included HbA1c as a continuous variable, and five studies (13, 14, 20, 41, 42) as a categorical variable. Due to high heterogeneity (I² = 92%, P < 0.001), a random-effects model was applied. Results from the meta-analysis of continuous variables suggested that lower HbA1c was associated with higher odds of remission (OR = 0.91, 95% CI = [0.88, 0.94], I² = 92% P < 0.001; **Figure 3C**). Subgroup analysis by T2DM remission criteria showed significant between-group differences (P < 0.001), but heterogeneity remained high within both subgroups (I² = 92%), suggesting that remission criteria alone did not fully explain the observed heterogeneity. Under strict remission definitions, the pooled OR was 0.68 (95% CI = [0.46, 1.01]), with substantial heterogeneity (I² = 92%, P < 0.001); the confidence interval crossed the null value, indicating no statistically significant association in this subgroup. Under the non-strict remission definition, the overall pooled OR was 0.95 (95% CI = [0.92, 0.98], I² = 92%, P < 0.001). Since the five included studies reporting categorical HbA1c used inconsistent thresholds (3 studies used < 7% vs. ≥ 7%, and 2 studies used < 8% vs. ≥ 8%), this study pooled the results of studies with different thresholds separately. The results showed that for the three studies using a 7% threshold (20, 41, 42), yielding an overall pooled OR was 2.98 (95% CI = [1.91, 4.64], I² = 63%, P < 0.001; **Figure 3D**). For the two studies using a threshold of 8% (13, 14), a fixed-effects model was applied due to low heterogeneity, yielding an overall pooled OR was 2.25 (95% CI = [1.50, 3.38], I² = 0%, P < 0.001; **Figure 3E**). The results from both categorical analyses were consistent with the continuous variable findings, collectively suggesting that lower HbA1c was associated with higher odds of T2DM remission.

#### 3.4.4 HOMA-IR

A total of two studies (21, 45) were included. Due to high heterogeneity, a random-effects model was used (I² = 83%, P = 0.02). The pooled analysis suggested that HOMA-IR was not significantly associated with T2DM remission (OR = 0.91, 95% CI = [0.72, 1.14], P = 0.400; **Figure 3F**). Furthermore, Salman et al. (36), patients were divided into two groups based on HOMA-IR: ≤ 4.6 and > 4.6. The results showed that patients with HOMA-IR ≤4.6 had higher remission odds (OR = 35.80, 95% CI = [9.60, 134.00], P < 0.001), although the confidence interval was wide, indicating limited precision.

#### 3.4.5 C-peptide level

A total of six studies (13, 22, 27, 35–37) were included. Among them, three studies (22, 35, 37) included C-peptide levels as a categorical variable, the results showed that C-peptide levels was not associated with T2DM remission (OR = 1.09, 95% CI = [0.97, 1.23], I² = 53%, P = 0.150; **Figure 3G**). The remaining three studies (13, 27, 36) also analyzed C-peptide as a categorical variable; however, differing classification criteria precluded a meta-analysis. Agha et al. (13) categorized C-peptide levels as >3 ng/mL and ≤3 ng/mL, and their results showed that high C-peptide levels were significantly associated with T2DM remission (OR = 2.67, 95% CI = [1.45, 4.91], P = 0.002). Salman et al. (36) grouped participants based on whether C-peptide levels exceeded 2.72 ng/mL. The results showed that higher C-peptide levels were associated with a higher rate of T2DM remission (OR = 282.20, 95% CI = [23.60, 3377.50], P < 0.001). Luo et al. (27) grouped participants based on whether C-peptide AUC was greater than 30.93, and also demonstrated a significant positive correlation (OR = 8.14, 95% CI = [1.96, 33.76], P = 0.002). All three studies consistently show that higher C-peptide levels are associated with higher odds of T2DM remission.

### 3.5 Disease-related and treatment factors

#### 3.5.1 Disease duration

A total of 21 studies (6, 13, 16, 18–23, 28–30, 34, 36, 37, 39, 42, 43) were included. Of these, 12 studies (6, 18, 21–23, 29, 30, 34, 37, 39, 42, 43) included disease duration as a continuous variable, six studies (13, 16, 19, 20, 28, 36) included as a categorical variable, and three studies (14, 40, 41) conducted qualitative descriptive analyses. Due to low heterogeneity (I² = 43%, P = 0.060), a fixed-effects model was used for the continuous variables. Shorter disease duration was associated with higher odds of remission (OR = 0.89, 95% CI = [0.88, 0.90], P < 0.001; **Figure 4A**). A fixed-effects model meta-analysis of categorical variables showed that patients with a diabetes duration of less than 5 years had a significantly higher T2DM remission rate than those with a duration of 5 years or longer (OR = 3.41, 95% CI = [2.35, 4.96], I² = 48%, P < 0.001; **Figure 4B**). Additionally, three other studies reported on the impact of duration on T2DM remission. Ahmad et al. (14) grouped patients based on whether their duration of T2DM was less than 8 years, a shorter duration was significantly associated with a higher remission rate (OR = 2.80, 95% CI = [1.50, 5.10], P = 0.002). Tripathi et al. (41) categorized duration into two groups: ≤ 6 years and > 6 years (OR = 3.48, 95% CI = [2.70, 4.48], P < 0.001). Thapa et al. (40) used ≥ 4 years as the reference group and compared duration of 1–4 years (OR = 1.59, 95% CI = [1.51, 1.67], P < 0.001) and < 1 year (OR = 2.59, 95% CI = [2.46, 2.73], P < 0.001) with remission of T2DM. The results indicate an association between shorter diabetes duration and higher odds of remission.

**Figure 4:**
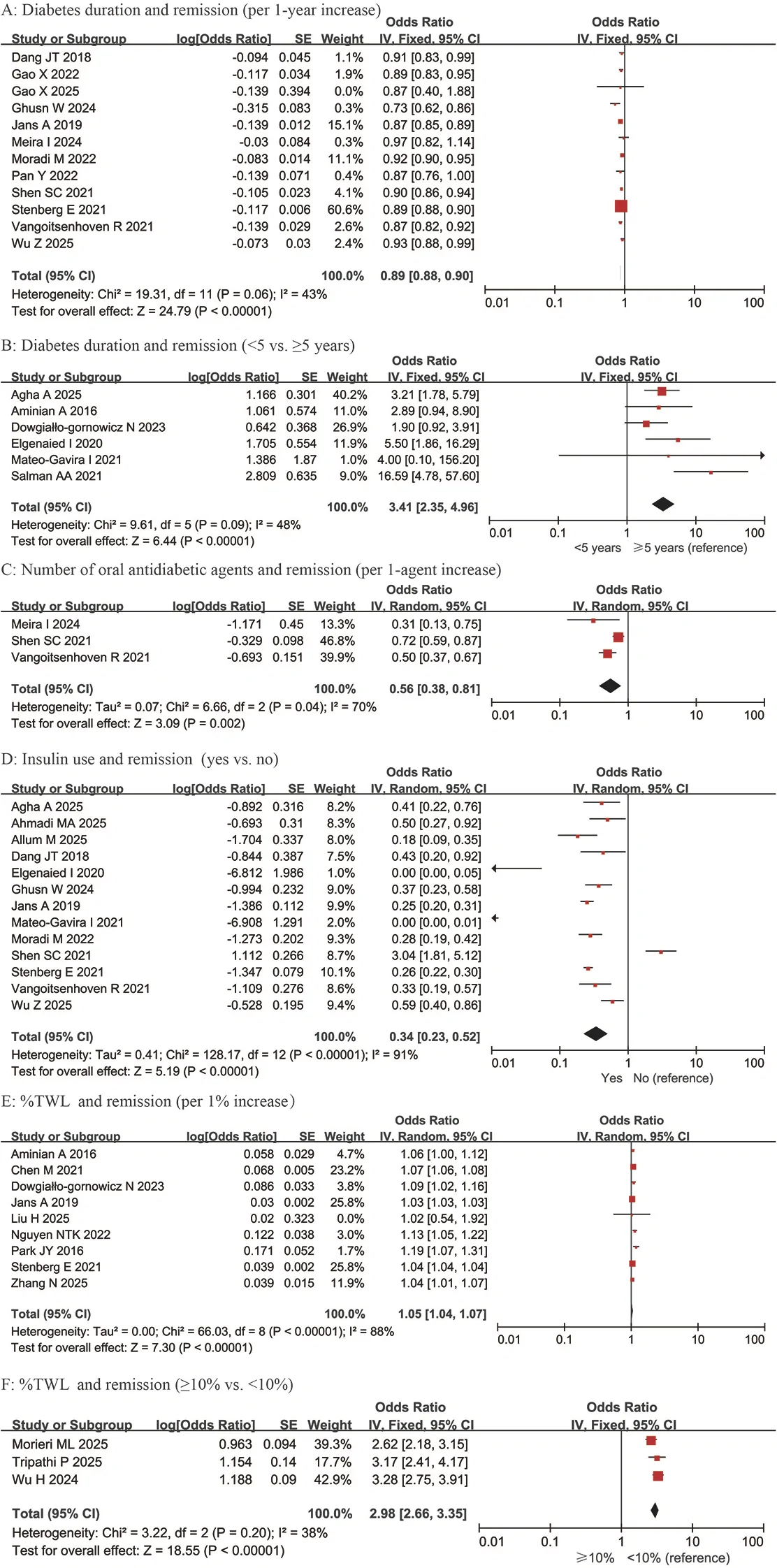
Forest Plot of Disease-Related and Treatment Factors. **(A)** Diabetes duration (per 1-year increase); **(B)** Diabetes duration (<5 vs. ≥5 years); **(C)** Number of oral antidiabetic agents; **(D)** Insulin use; **(E)** %TWL (per 1% increase); **(F)** %TWL (≥10% vs. <10%).

#### 3.5.2 Number of oral antidiabetic agents

A total of six studies (29, 30, 36, 37, 40, 42) evaluated the association between the number of oral antidiabetic agents and T2DM remission. Of these, three studies were included in the continuous variable meta-analysis, and the remaining three studies were described qualitatively. Due to substantial heterogeneity (I² = 70%, P = 0.04), a random-effects model was used. The continuous variables analysis showed that fewer oral antidiabetic agents was associated with higher odds of remission (OR = 0.56, 95% CI = [0.38, 0.81], P = 0.002; **Figure 4C**). The remaining three studies also evaluated this association; however, due to differences in reference groups and drug classification criteria across studies, a meta-analysis could not be performed. Thapa et al. (40) found that, when using ≥ 3 antidiabetic drugs as the reference group, both 1 drug (OR = 6.35, 95% CI = [4.84, 8.34], P < 0.001) and 2 drugs (OR = 2.77, 95% CI = [2.10, 3.66], P < 0.001) were significantly associated with remission. Salman et al. (36) compared the effects of monotherapy versus combination therapy with oral hypoglycemic agents and found that a smaller number of hypoglycemic agents was associated with a higher remission odds (OR = 8.00, 95% CI = [1.80, 35.40], P = 0.006). However, some studies have reached the opposite conclusion. In contrast, Moradi et al. (30) reported that the number of oral antidiabetic agents was not significantly associated with T2DM remission (P > 0.05).

#### 3.5.3 Insulin use

A total of 13 studies (6, 13–15, 18, 20, 23, 28, 30, 37, 39, 42, 44) evaluated the association between insulin use and remission of T2DM. Due to high heterogeneity (I² = 91%, P < 0.001), a random-effects model was used. Heterogeneity exploration showed insulin use was associated with lower odds of remission (OR = 0.34, 95% CI = [0.23, 0.52], P < 0.001; **Figure 4D**). Subgroup analysis by T2DM remission criteria showed significant between-group differences (P = 0.008). Heterogeneity was substantially reduced in the strict definition subgroup (I² = 61%) but remained high in the non-strict definition subgroup (I² = 94%). Under the strict definition of remission, the overall pooled OR = 0.39 (95% CI = [0.27, 0.58], I² = 61%, P = 0.036); under the non-strict remission definition, the overall pooled OR = 0.30 (95% CI = [0.17, 0.55]), with persistently high heterogeneity (I² = 94%, P < 0.001), suggesting that this estimate is less reliable.

#### 3.5.4 Percentage of total weight loss

A total of 14 studies (5, 6, 13, 16, 19, 20, 26, 31, 32, 35, 39, 41, 43, 45) were included. Of these, nine studies (5, 6, 16, 19, 26, 32, 35, 39, 45) included %TWL as a continuous variable, three studies (31, 41, 43) included as a categorical variable, and two studies (13, 20) were described qualitatively. Due to high heterogeneity (I² = 88%, P < 0.001), a random-effects model was applied. The continuous variable analysis indicated that a higher %TWL was associated with higher odds of remission (OR = 1.05, 95% CI = [1.04, 1.07], P < 0.001; **Figure 4E**). When subgroup analyses were conducted based on intervention type and T2DM remission criteria, the level of heterogeneity decreased significantly, suggesting that these two factors may be the causes of the heterogeneity. For surgical studies, the overall pooled OR was 1.04 (95% CI = [1.03, 1.06], I² = 74%, P < 0.001), whereas for the non-surgical studies the overall pooled OR = 1.07 (95% CI = [1.06, 1.08], I² = 0%, P = 0.880). Further subgroup analyses were performed by specific surgical procedure. For sleeve gastrectomy, the pooled OR was 1.09 (95% CI = [1.02, 1.16], I² = 44%, P = 0.007); for Roux-en-Y gastric bypass, the pooled OR was 1.10 (95% CI = [0.97, 1.25], I² = 85%, P = 0.144); and for mixed bariatric procedures, the pooled OR was 1.04 (95% CI = [1.02, 1.06], I² = 40%, P < 0.001). The between-group difference was statistically significant (P = 0.003), suggesting that the association between %TWL and remission differed across procedure types. Subgroup analysis based on T2DM remission criteria showed that under the strict remission definition, the overall pooled OR was 1.04 (95% CI = [1.02, 1.07], I² = 0%, P = 0.557). Under a non-strict remission definition, the overall pooled OR = 1.06 (95% CI = [1.04, 1.07], I² = 91%, P < 0.001). A fixed-effects meta-analysis of categorical variables showed that %TWL ≥ 10% was associated with higher odds of remission (OR = 2.98, 95% CI = [2.66, 3.35], I² = 38%, P < 0.001; **Figure 4F**). Additionally, Agha et al. (13) reported that patients with a %TWL of ≥ 20% at 1 year post-surgery had a significantly higher T2DM remission odds than those with a weight loss of < 20% (OR = 4.15, 95% CI = [2.23, 7.72], P < 0.001). Elgenaied et al. (20) used %TWL > 50% as the cutoff and found that this threshold was significantly associated with a higher remission odds (OR = 2.39, 95% CI = [1.12, 5.12], P = 0.025).

### 3.6 Sensitivity analysis

Sensitivity analysis was conducted using the stepwise elimination method for relevant factors that were included in ≥ 3 studies and exhibited high heterogeneity. All factors showed good consistency, suggesting that the results of this study are generally reliable (**Supplementary Figure 1**). Sensitivity analyses comparing fixed-effects and random-effects models were performed for key factors (**Supplementary Table 5**). For age, diabetes duration, HbA1c, BMI, number of oral antidiabetic agents, insulin use, and %TWL, the direction and statistical significance were consistent across both models, supporting the robustness of these associations. For fasting plasma glucose and smoking, estimates were also consistent across models, with low heterogeneity. In contrast, gender, race/ethnicity, and physical exercise showed different statistical significance between the two models, indicating that these estimates were not robust to model choice. Notably, gender, race/ethnicity, and physical exercise were not significantly associated with T2DM remission in the random-effects model, and the apparent significance under the fixed-effects model likely reflects high heterogeneity rather than a true association. For HOMA-IR and C-peptide, neither model showed a significant association.

### 3.7 Assessment of publication bias

Using Stata 18.0 software, Egger’s test was performed on factors with ≥10 included studies to assess publication bias. The results indicated that publication bias may exist for HbA1c (**Supplementary Table 6**). A funnel plot also revealed overall asymmetry (**Supplementary Figure 1**). The results for the remaining factors were favorable, with generally symmetrical funnel plots, suggesting no significant publication bias. For factors showing significant publication bias, the trim-and-fill method was used for assessment. The results indicated that the trim-and-fill method identified 4 theoretically missing studies. Before adjustment, the pooled OR = 0.74 (95% CI = [0.64, 0.86], P < 0.001). After adjustment, the pooled OR = 0.84 (95% CI = [0.72, 0.98], P = 0.029), indicating that the pooled results of the meta-analysis are relatively stable.

Several factors may explain the publication bias observed for baseline HbA1c. First, selective reporting may have contributed. Studies reporting a strong inverse association between HbA1c and remission are more likely to be published, whereas studies with null or weak findings may remain unpublished or be reported incompletely. Second, small-study effects may be present. Smaller studies often report larger effect estimates, which may arise from lower methodological quality, greater heterogeneity in patient selection, or the fact that they are more likely to be conducted in specific populations. Third, the trim-and-fill analysis identified only 4 missing studies, and the adjusted estimate remained statistically significant, suggesting that the observed bias is unlikely to overturn the overall conclusion.

## 4 Discussion

This meta-analysis evaluated the influencing factors of T2DM remission. The results showed that younger age, lower fasting plasma glucose, lower glycated hemoglobin, shorter disease duration, fewer oral antidiabetic agents, and a higher percentage of total weight loss were associated with higher odds of remission, with consistent effects observed in both the surgical and non-surgical groups. Notably, the direction of BMI’s effect on T2DM remission was inconsistent between the two groups: among patients who underwent surgery, a higher BMI was associated with a higher probability of remission; whereas among non-surgical patients, a higher BMI was associated with a lower probability of remission. Furthermore, gender, race, physical exercise, intermittent fasting, HOMA-IR, and C-peptide levels did not appear to affect diabetes remission. Nevertheless, a complete exclusion of these factors is not achievable through this meta-analysis, given that many investigations have already established a link between them and the remission of T2DM (13, 35, 36, 41).

This study found that younger age, shorter disease duration, lower FPG, and lower HbA1c were all associated with higher remission rates, consistent with previous studies (9). The results further demonstrate that these factors apply not only to patients undergoing surgery but also to those receiving non-surgical interventions. Younger patients and those with a shorter disease duration are typically in the early stages, with milder long-term exposure to glucotoxicity and lipotoxicity, less severe β-cell dysfunction, and retained insulin secretion capacity, therefore, they are more likely to achieve remission following intervention (46, 47). A higher HbA1c and FPG levels indicate impaired β-cell function and reduced insulin sensitivity (48). This study have further demonstrated that a lower number of oral antidiabetic drugs and the absence of insulin use are both associated with higher remission rates. Conversely, the use of multiple oral antidiabetic drugs or insulin therapy may signify more severe and difficult-to-control diabetes, thereby conferring a lower likelihood of remission (49).

The opposite effects of BMI across different intervention modalities represent one of the most notable findings of this study. In surgical interventions, a higher BMI was associated with a higher remission rate for T2DM; conversely, in non-surgical interventions, a higher BMI correlated with a lower remission rate. Subgroup analysis showed a significant between-group difference (P < 0.001), suggesting that the predictive value of baseline BMI for T2DM remission may be modified by intervention type. However, this finding should be interpreted with caution. First, because only two studies were included in the non-surgical subgroup, formal interaction testing between intervention type and BMI could not be performed. Second, confounding by indication may partly explain the contrasting directions of effect. In clinical practice, patients with higher BMI are more likely to undergo bariatric surgery, whereas those with lower BMI tend to receive non-surgical interventions, potentially leading to systematic differences in baseline characteristics between the two groups. Such selection may generate an apparent inverse association between BMI and remission in non-surgical cohorts. Third, BMI itself may not be a direct determinant of remission but may operate indirectly by influencing the potential for weight loss and metabolic improvement: patients with higher BMI achieve greater weight loss and more significant metabolic improvements in surgical interventions (50, 51), which may lead to higher remission rates; by contrast, in non-surgical interventions, patients with higher BMI face greater difficulty in losing weight, resulting in lower remission rates. Thus, without sufficient weight loss, higher BMI itself does not confer a higher remission rate. Fourth, metabolic reserve and illness duration may also contribute to the divergent associations. In surgical cohorts, despite more severe baseline metabolic disturbances, a shorter disease duration and a stronger metabolic reversal after weight loss may still permit favorable remission outcomes. By contrast, patients receiving non-surgical management tend to have longer diabetes duration and reduced beta-cell reserve, and higher BMI limits glycemic improvement. Overall, the contrasting BMI effects may reflect differences in baseline clinical selection, weight-loss dynamics, and metabolic severity rather than a divergent intrinsic effect of BMI. Future studies with individual patient-level data and standardized eligibility criteria are needed to clarify whether the opposing BMI effects reflect a true effect modification or are largely driven by confounding by indication.

Notably, the analysis of %TWL further suggested that, regardless of surgical or non-surgical interventions, greater %TWL was significantly associated with higher odds of remission, and patients with %TWL ≥ 10% had a substantially increased likelihood of achieving remission. Further subgroup analyses by specific surgical procedure showed that the association between %TWL and remission varied across procedure types: sleeve gastrectomy and mixed procedures both showed a significant association between %TWL and remission, whereas Roux-en-Y gastric bypass did not reach statistical significance, possibly due to its small sample size (only 2 studies), suggesting that the association between %TWL and remission in Roux-en-Y gastric bypass warrants further investigation. The DiRECT study confirmed that significant weight loss achieved through a very low-calorie diet intervention enabled 46% of patients with early-stage T2DM to achieve remission at 12 months; among patients who lost ≥15 kg, the remission rate reached 86% (2). The 5-year follow-up results of this trial further confirmed that long-term remission is closely associated with weight maintenance (52). The “twin cycle hypothesis” proposed by Taylor et al. posits that excessive fat in the liver and pancreas can lead to β-cell dysfunction, whereas weight loss reduces ectopic fat in these organs and promotes β-cell function recovery, thereby restoring blood glucose to normal levels (53). Taken together, these findings suggest that achieved weight loss may be the key factor in achieving remission. Regardless of whether surgical or non-surgical approaches are employed, any intervention that achieves adequate and sustained weight loss may improve the probability of T2DM remission. These results suggest that bariatric surgery may be a more favorable option for achieving T2DM remission in patients with higher BMI, whereas intensive lifestyle modification and pharmacotherapy may be more effective in achieving disease remission for patients with lower BMI or those who are unsuitable for surgery.

Among non-surgical interventions, abstinence from alcohol is a contributing factor to remission of T2DM, whereas continued alcohol consumption and smoking were associated with lower odds of remission. Long-term smoking can induce oxidative stress and inflammatory responses, reduce the body’s insulin sensitivity, promote insulin resistance and lead to elevated blood glucose levels (54, 55). These findings reinforce current guidance that smoking cessation is strongly recommended for individuals with T2DM, as it may improve overall health and could also support diabetes remission. Previous studies have shown (56) that higher alcohol consumption increases the risk of developing T2DM, indicating that alcohol consumption affects glucose metabolism. At the same time, the findings support that alcohol cessation was associated with higher odds of remission. In contrast, physical activity and intermittent fasting did not show significant associations, which may be related to the small number of included studies, or may suggest that the independent contribution of a single behavioral intervention to remission is limited if it does not result in sufficient weight loss.

HOMA-IR reflects the degree of insulin resistance, while C-peptide levels reflect insulin secretion capacity and pancreatic β-cell reserve. Both are closely associated with T2DM remission. However, no association was observed in the meta-analysis between HOMA-IR and C-peptide levels and T2DM remission. This may be because the relatively small number of studies reporting HOMA-IR and C-peptide, together with the heterogeneity in C-peptide assays across studies, limited quantitative pooling. Nevertheless, qualitative findings highlight its clinical value. Lee et al. (57) found that baseline C-peptide levels could predict T2DM remission following bariatric surgery. Salman et al. (36) found in patients undergoing sleeve gastrectomy that lower HOMA-IR and higher C-peptide levels were predictors of T2DM remission. Therefore, although this study did not find a significant association between HOMA-IR and C-peptide levels and T2DM remission due to the limitations of meta-analytic pooling, this does not negate their potential predictive value, and baseline β-cell reserve remains a critical marker.

This study offers multiple strong points. First, we systematically summarized the factors influencing T2DM remission following surgical and non-surgical interventions, with a particular focus on the opposite effects of BMI across different intervention types. Second, we identified key influencing factors thresholds, including %TWL ≥10%, HbA1c < 7%, and disease duration < 5 years; these thresholds are clearly clinically actionable. Third, this study covers a comprehensive range of factors, including patient-related factors (gender, age, race/ethnicity, lifestyle factors), metabolic and laboratory parameters (BMI, fasting plasma glucose, HbA1c, HOMA-IR, C-peptide level), and disease-related and treatment factors (disease duration, number of oral antidiabetic agents, insulin use, %TWL).

However, this study also has limitations. First, the number of available studies on non-surgical interventions prevented subgroup analyses based on intervention type for some factors, and some subgroup analyses included only a small number of studies, which precluded formal testing of the interaction between intervention type and the influencing factors. All subgroup findings based on small study counts should therefore be considered exploratory, and conclusions drawn from these subgroups require cautious interpretation and confirmation in larger prospective studies. Second, for the continuous variables, substantial heterogeneity was observed for both HbA1c and insulin use. Although we conducted subgroup analyses based on remission criteria, this did not significantly reduce the overall heterogeneity, which may be attributable to across-study differences in baseline patient characteristics, intervention protocols, follow-up durations, and timing of remission assessment. Third, for the categorical variable analyses, the cut-off points varied considerably across studies, with only certain thresholds having sufficient studies to support independent pooling, which may have introduced classification bias and limited the reliability of the conclusions. Furthermore, our study exclusively included patients with established T2DM and did not investigate prediabetes remission. Prediabetes remission refers to the restoration of metabolic function at an early stage of dysglycemia, with its primary advantage being that interventions at this stage can prevent progression to T2DM. Recent evidence indicates that achieving prediabetes remission confers significant cardiovascular protection, sustainably reducing the risk of cardiovascular death and heart failure (58). These findings suggest that the cardiovascular benefits derived from metabolic recovery may depend on the stage of dysglycemia at which this state is achieved. Given that our study population was strictly limited to patients with established T2DM, our conclusions should not be extrapolated to the prediabetes population, nor can we directly compare the magnitude of cardiovascular protection between these two remission states. Future high-quality, large-scale, prospective studies are needed to further validate these findings.

## Data Availability

The original contributions presented in the study are included in the article/**Supplementary Material**. Further inquiries can be directed to the corresponding author.
