# Supplementary material for: Influencing factors of type 2 diabetes remission: a systematic review and meta-analysis
Source: Front Endocrinol (Lausanne). 2026 Oct 15;17:1916734. doi: 10.3389/fendo.2026.1916734 (PMC13716553; doi:10.3389/fendo.2026.1916734)
Supplement: Supplementary file 1 [file SupplementaryFile1.docx]

Supplementary Material

**Supplementary Table 1.** Search Strategy and Detailed Search Term

| **Database** | **Search terms** | **Results** |
| --- | --- | --- |
| Web of Science | #1 TS=(Diabetes Mellitus, Type 2 OR non-insulin-dependent diabetes OR type 2 diabetes OR type 2 diabetes mellitus T2DM OR diabetes type 2 OR type II diabetes OR type 2 diabetes OR non insulin dependent diabetes OR NIDDM OR type 2 diabetes mellitus OR adult onset diabetes OR maturity onset diabetes)  #2 TS=(remiss* OR resolution OR normoglycemi* OR revers* OR drug-free OR glycemic remission OR sustained normoglycemia OR diabetes remission OR partial remission OR complete remission)  #3 TS=( risk factor* OR prognosis OR prognostic factor* OR influencing factor* OR predict* OR determinant* OR associated factor* OR contributing factors )  #4 #1 AND #2 AND #3 6146  #5 #4 AND PY=(2009-2026) 5099 | 5099 |
| PubMed | #1 "Diabetes Mellitus, Type 2"[MeSH Terms] OR "non-insulin-dependent diabetes"[Title/Abstract] OR "diabetes mellitus, type 2"[Title/Abstract] OR "type 2 diabetes mellitus"[Title/Abstract] OR "T2DM"[Title/Abstract] OR "diabetes type 2"[Title/Abstract] OR "type II diabetes"[Title/Abstract] OR "type 2 diabetes"[Title/Abstract] OR "non insulin dependent diabetes"[Title/Abstract] OR "NIDDM"[Title/Abstract] OR "adult onset diabetes"[Title/Abstract] OR "maturity onset diabetes"[Title/Abstract]  #2 "remiss*"[Title/Abstract] OR "resolution"[Title/Abstract] OR "normoglycemi*"[Title/Abstract] OR "revers*"[Title/Abstract] OR "drug-free"[Title/Abstract] OR "glycemic remission"[Title/Abstract] OR "sustained normoglycemia"[Title/Abstract] OR "diabetes remission"[Title/Abstract] OR "partial remission"[Title/Abstract] OR "complete remission"[Title/Abstract]  #3 "Risk Factors"[MeSH Terms] OR "Prognosis"[MeSH Terms] OR "risk factor*"[Title/Abstract] OR "influencing factor*"[Title/Abstract] OR "predict*"[Title/Abstract] OR "determinant*"[Title/Abstract] OR "associated factor*"[Title/Abstract] OR "contributing factor*"[Title/Abstract] OR "prognostic factor*"[Title/Abstract]  #4 #1 AND #2 AND #3 4675  "2009"[Date - Publication] : "2026"[Date - Publication] 3986 | 3986 |
| Embase | #1 'non insulin dependent diabetes mellitus'/exp OR 'non-insulin-dependent diabetes':ab,ti OR 'diabetes mellitus, type 2':ab,ti OR 'type 2 diabetes mellitus':ab,ti OR 't2dm':ab,ti OR 'diabetes type 2':ab,ti OR 'type ii diabetes':ab,ti OR 'type 2 diabetes':ab,ti OR 'non insulin dependent diabetes':ab,ti OR 'niddm':ab,ti OR 'adult onset diabetes':ab,ti OR 'maturity onset diabetes':ab,ti  #2 'remiss*':ab,ti OR 'resolution':ab,ti OR 'normoglycemi*':ab,ti OR 'revers*':ab,ti OR 'drug-free':ab,ti OR 'glycemic remission':ab,ti OR 'sustained normoglycemia':ab,ti OR 'diabetes remission':ab,ti OR 'partial remission':ab,ti OR 'complete remission':ab,ti  #3 'risk factor'/exp OR 'prognosis'/exp OR 'risk factor*':ab,ti OR 'influencing factor*':ab,ti OR 'predict*':ab,ti OR 'determinant*':ab,ti OR 'associated factor*':ab,ti OR 'contributing factor*':ab,ti OR 'prognostic factor*':ab,ti  #4 #1 AND #2 AND #3 6699  #5 #4 AND [2009-2026]/py 6016 | 6016 |
| Cochrane | #1 MeSH descriptor: [Diabetes Mellitus, Type 2] explode all trees  #2 (non-insulin-dependent diabetes):ti,ab,kw OR(diabetes mellitus, type 2):ti,ab,kw OR (type 2 diabetes mellitus):ti,ab,kw OR (T2DM):ti,ab,kw OR (diabetes type 2):ti,ab,kw OR (type II diabetes):ti,ab,kw OR (type 2 diabetes):ti,ab,kw OR (non insulin dependent diabetes):ti,ab,kw OR (NIDDM):ti,ab,kw OR (adult onset diabetes):ti,ab,kw OR (maturity onset diabetes):ti,ab,kw  #3 #1 OR #2  #4 (remiss*):ti,ab,kw OR (resolution):ti,ab,kw OR (normoglycemi):ti,ab,kw OR (revers*):ti,ab,kw OR (drug-free):ti,ab,kw OR (glycemic remission):ti,ab,kw OR (sustained normoglycemia):ti,ab,kw OR (diabetes remission):ti,ab,kw OR (partial remission):ti,ab,kw OR (complete remission):ti,ab,kw  #5 MeSH descriptor: [Risk Factors] explode all trees  #6 MeSH descriptor: [Prognosis] explode all trees  #7 (risk factor*):ti,ab,kw OR (influencing factor*):ti,ab,kw OR (predict*):ti,ab,kw OR (determinant*):ti,ab,kw OR (associated factor*):ti,ab,kw OR (contributing factor*):ti,ab,kw OR (prognostic factor*):ti,ab,kw  #8 #5 OR #6 OR #7  #9 #3 AND #4 AND #8 1014  with Publication Year from 2009 to 2026, in Trials 879 | 879 |

**Supplementary Table 2.** Definitions of T2DM in the Included Studies

| Study | Remission Type | HbA1c Cutoff | FPG Cutoff | Medication Withdrawal | Duration | Classification |
| --- | --- | --- | --- | --- | --- | --- |
| Agha A 2025 | Complete | < 6.0% | Not specified | Yes | ≥ 1 year | Strict |
|  | Partial | < 6.5% | Not specified | Yes | ≥ 1 year | Strict |
| Ahmadi MA 2025 | Complete | < 5.7% | < 100 mg/dL | Yes | ≥ 1 year | Strict |
|  | Partial | < 6.5% | 100-125mg/dL | Yes | ≥ 1 year | Strict |
| Allum M 2025 | Remission | < 6.5% | Not specified | Yes | ≥ 3 months | Strict |
| Aminian A 2016 | Remission | < 6.5% | < 126 mg/dL | Yes | ≥ 5 years | Strict |
| Chen M 2021 | Remission | < 6.5% | Not specified | Not specified | Not specified | Non-strict |
| Coelho D 2018 | Complete | < 6.0% | < 100 mg/dL | Yes | Not specified | Non-strict |
|  | Partial | < 6.5% | < 125 mg/dL | Yes | Not specified | Non-strict |
| Dang JT 2018 | Remission | < 6.5% | Not specified | Yes | Not specified | Non-strict |
| Dogaiello-gornowicz N 2023 | Remission | < 6.0% | < 100 mg/dL | Yes | Not specified | Non-strict |
| Elgenaied I 2020 | Complete | < 6.0% | < 100 mg/dL | Yes | Not specified | Non-strict |
|  | Partial | 6-6.4% | 100–125 mg/dL | Yes | Not specified | Non-strict |
| Gao X 2022 | Remission | < 6.0% | ≤ 5.6 mmol/L | Yes | ≥ 12 months | Strict |
| Gao X 2025 | Complete | < 6.0% | < 6.9 mmol/L | Yes | ≥ 12 months | Strict |
|  | Partial | < 6.5% | < 5.5 mmol/L | Yes | ≥ 12 months | Strict |
| Ghusn W 2024 | Remission | < 6.5% | Not specified | Yes | Not specified | Non-strict |
| Giudicelli G 2026 | Remission | < 6.0% | < 5.55 mmol/L | Yes | Not specified | Non-strict |
| Jans A 2019 | Remission | < 6.0% | Not specified | Yes | Not specified | Non-strict |
| Kim J 2025 | Remission | Not specified | < 126 mg/dL | Yes | ≥ 2 years | Non-strict |
| Liu H 2025 | Remission | < 6.5% | Not specified | Not specified | ≥ 6 months | Non-strict |
| Luo Y 2020 | Remission | < 6.5% | < 7.1 mmol/L | Yes | ≥ 1 year | Strict |
| Mateo-Gavira I 2021 | Remission | < 6.5% | < 6.99 mmol/L | Yes | Not specified | Non-strict |
| Matsui T 2023 | Remission | Not specified | < 126 mg/dL | Yes | Not specified | Non-strict |
| Meira I 2024 | Complete | < 5.7% | < 100 mg/dL | Yes | Not specified | Non-strict |
|  | Partial | 5.7–6.5% | 100–125 mg/dL | Yes | Not specified | Non-strict |
| Moradi M 2022 | Complete | < 6.0% | < 100 mg/dL | Yes | Not specified | Non-strict |
|  | Partial | 6.0–6.4% | 100–125 mg/dL | Yes | Not specified | Non-strict |
| Morieri ML 2025 | Remission | < 6.5% | Not specified | Yes | ≥ 90 days | Strict |
| Nguyen NTK 2022 | Remission | < 5.7% | < 100 mg/dL | Yes | Not specified | Non-strict |
| Niu L 2024 | Complete | < 6.0% | < 5.6 mmol/L | Yes | ≥ 12 months | Strict |
|  | Partial | < 6.5% | 5.6–6.9 mmol/L | Yes | ≥ 12 months | Strict |
| Pan Y 2022 | Remission | < 6.0% | < 5.6 mmol/L | Yes | ≥ 1 year | Strict |
| Park JY 2016 | Complete | < 6.0% | Not specified | Yes | Not specified | Non-strict |
|  | Partial | < 6.5% | Not specified | Yes | Not specified | Non-strict |
| Salman AA 2021 | Complete | < 6.0% | < 100 mg/dL | Yes | Not specified | Non-strict |
|  | Partial | 6.0–6.4% | 100–125 mg/dL | Yes | Not specified | Non-strict |
| Shen SC 2021 | Remission | < 6.0% | Not specified | Yes | Not specified | Non-strict |
| Silva MM 2022 | Complete | < 6.0% | Not specified | Yes | Not specified | Non-strict |
|  | Partial | < 6.5% | Not specified | Yes | Not specified | Non-strict |
| Stenberg E 2021 | Remission | Not specified | Not specified | Yes | ≥ 2 years | Non-strict |
| Thapa B 2025 | Remission | < 6.5% | Not specified | Yes | ≥ 3 months | Strict |
| Tripathi P 2025 | Remission | < 6.5% | Not specified | Yes | ≥ 3 months | Strict |
| Vangoishenoven R 2021 | Remission | < 6.5% | Not specified | Yes | ≥ 5 years | Strict |
| Wu H 2024 | Remission | < 6.5% | Not specified | Yes | ≥ 3 months | Strict |
| Wu Z 2025 | Remission | < 6.5% | Not specified | Yes | ≥ 3 months | Strict |
| Zhang N 2025 | Remission | < 6.5% | Not specified | Yes | ≥ 3 months | Strict |

**Supplementary Table 3.** Quality Assessment of Included Studies

| Study | Selection | | Comparability | Outcome | Total score |
| --- | --- | --- | --- | --- | --- |
| Agha A 2025 | *** | ** | | *** | 8 |
| Ahmadi MA 2025 | *** | ** | | *** | 8 |
| Allum M 2025 | *** | ** | | ** | 7 |
| Aminian A 2016 | ** | ** | | *** | 7 |
| Chen M 2021 | **** | ** | | *** | 9 |
| Coelho D 2018 | *** | ** | | ** | 7 |
| Dang JT 2018 | *** | ** | | *** | 8 |
| Dowgiałło-gornowicz N 2023 | *** | ** | | *** | 8 |
| Elgenaied I 2020 | ** | ** | | *** | 7 |
| Gao X 2022 | *** | ** | | *** | 8 |
| Gao X 2025 | *** | ** | | *** | 8 |
| Ghusn W 2024 | **** | ** | | *** | 9 |
| Giudicelli G 2026 | **** | ** | | *** | 9 |
| Jans A 2019 | **** | ** | | ** | 8 |
| Kim J 2025 | **** | ** | | *** | 9 |
| Liu H 2025 | ** | ** | | *** | 7 |
| Luo Y 2020 | **** | ** | | ** | 8 |
| Mateo-Gavira I 2021 | *** | ** | | *** | 8 |
| Matsui T 2023 | **** | ** | | ** | 8 |
| Meira I 2024 | *** | ** | | ** | 7 |
| Moradi M 2022 | *** | ** | | ** | 7 |
| Morieri ML 2025 | **** | ** | | ** | 8 |
| Nguyen NTK 2022 | *** | ** | | *** | 8 |
| Niu L 2024 | *** | ** | | *** | 8 |
| Pan Y 2022 | *** | ** | | *** | 8 |
| Park JY 2016 | *** | ** | | ** | 7 |
| Salman AA 2021 | *** | ** | | *** | 8 |
| Shen SC 2021 | **** | ** | | ** | 8 |
| Silva MM 2022 | *** | ** | | *** | 8 |
| Stenberg E 2021 | **** | ** | | *** | 9 |
| Thapa B 2025 | **** | ** | | *** | 9 |
| Tripathi P 2025 | *** | ** | | *** | 8 |
| Vangoitsenhoven R 2021 | *** | ** | | ** | 7 |
| Wu H 2024 | **** | ** | | *** | 9 |
| Wu Z 2025 | **** | ** | | ** | 8 |
| Zhang N 2025 | *** | ** | | ** | 7 |

| **Supplementary Table 4.** Subgroup Analysis of Included Studies | | | | | | |
| --- | --- | --- | --- | --- | --- | --- |
| Factors | Subgroup | OR | 95%CI | I^2^ | No. studies | P for interaction |
| Gender | T2DM remission criteria |  |  |  |  | 0.007 |
|  | Strict remission definition | 1.10 | 0.99,1.23 | 63% | 2 |  |
|  | Non-strict remission definition | 0.79 | 0.64,0.98 | 75% | 6 |  |
|  | Type of intervention |  |  |  |  | 0.280 |
|  | Surgical intervention | 1.01 | 0.77,1.33 | 93% | 6 |  |
|  | Non-surgical intervention | 0.82 | 0.64,1.05 | 81% | 2 |  |
| Age | T2DM remission criteria |  |  |  |  | 0.001 |
|  | Strict remission definition | 0.96 | 0.93,0.98 | 44% | 2 |  |
|  | Non-strict remission definition | 0.96 | 0.94,0.98 | 97% | 9 |  |
|  | Type of intervention |  |  |  |  | ＜0 .001 |
|  | Surgical intervention | 0.95 | 0.94,0.97 | 81% | 9 |  |
|  | Non-surgical intervention | 0.99 | 0.95,1.03 | 94% | 2 |  |
| BMI | T2DM remission criteria |  |  |  |  | ＜0 .001 |
|  | Strict remission definition | 1.07 | 1.03,1.11 | 53% | 4 |  |
|  | Non-strict remission definition | 1.02 | 0.98,1.07 | 93% | 10 |  |
|  | Type of intervention |  |  |  |  | ＜0 .001 |
|  | Surgical intervention | 1.05 | 1.02,1.08 | 88% | 12 |  |
|  | Non-surgical intervention | 0.89 | 0.86,0.92 | 0% | 2 |  |
|  | Specific surgical procedure |  |  |  |  | ＜0 .001 |
|  | Roux-en-Y gastric bypass | 1.07 | 1.00,1.14 | 91% | 4 |  |
|  | Mixed procedures | 1.04 | 1.01,1.08 | 79% | 8 |  |
| HbA1c | T2DM remission criteria |  |  |  |  | ＜0 .001 |
|  | Strict remission definition | 0.68 | 0.46,1.01 | 92% | 3 |  |
|  | Non-strict remission definition | 0.95 | 0.92,0.98 | 92% | 7 |  |
| Insulin use | T2DM remission criteria |  |  |  |  | 0.008 |
|  | Strict remission definition | 0.39 | 0.27,0.58 | 61% | 5 |  |
|  | Non-strict remission definition | 0.30 | 0.17,0.55 | 94% | 8 |  |
| %TWL | T2DM remission criteria |  |  |  |  | 0.742 |
|  | Strict remission definition | 1.04 | 1.02,1.07 | 0% | 2 |  |
|  | Non-strict remission definition | 1.06 | 1.04,1.07 | 91% | 7 |  |
|  | Type of intervention |  |  |  |  | ＜0 .001 |
|  | Surgical intervention | 1.04 | 1.03,1.06 | 74% | 7 |  |
|  | Non-surgical intervention | 1.07 | 1.06,1.08 | 0% | 2 |  |
|  | Specific surgical procedure |  |  |  |  | 0.003 |
|  | sleeve gastrectomy | 1.09 | 1.02,1.16 | 44% | 2 |  |
|  | Roux-en-Y gastric bypass | 1.10 | 0.97,1.25 | 85% | 2 |  |
|  | Mixed procedures | 1.04 | 1.02,1.06 | 40% | 3 |  |

**Supplementary Table 5.** Sensitivity Analysis Comparing Fixed-effects and Random-effects Models

| Factors | Model | OR | 95% CI | P value | I^2^ |
| --- | --- | --- | --- | --- | --- |
| Gender | Fixed effects model | 1.09 | 1.06,1.13 | <0.001 | 90% |
|  | Random effects model | 0.88 | 0.73,1.07 | 0.20 | 90% |
| Age (per 1-year increase) | Fixed effects model | 0.98 | 0.97,0.98 | <0.001 | 95% |
|  | Random effects model | 0.96 | 0.94,0.98 | <0.001 | 95% |
| Age (≤45 vs. >45 years) | Fixed effects model | 2.39 | 1.58,3.63 | 0.001 | 46% |
|  | Random effects model | 2.62 | 1.43,4.79 | 0.002 | 46% |
| Race/Ethnicity | Fixed effects model | 1.20 | 1.14,1.26 | <0.001 | 94% |
|  | Random effects model | 1.05 | 0.75,1.47 | 0.76 | 94% |
| Smoking | Fixed effects model | 0.90 | 0.85,0.94 | < 0.001 | 0% |
|  | Random effects model | 0.90 | 0.85,0.94 | < 0.001 | 0% |
| Physical exercise | Fixed effects model | 0.66 | 0.62,0.70 | <0.001 | 84% |
|  | Random effects model | 0.75 | 0.54,1.04 | 0.080 | 84% |
| BMI | Fixed effects model | 1.02 | 1.01,1.02 | <0.001 | 92% |
|  | Random effects model | 1.04 | 1.00,1.07 | 0.050 | 92% |
| Fasting Plasma Glucose | Fixed effects model | 0.99 | 0.99,1.00 | <0.001 | 19% |
|  | Random effects model | 0.99 | 0.99,1.00 | <0.001 | 19% |
| HbA1c (per 1% increase) | Fixed effects model | 0.98 | 0.98,0.99 | < 0.001 | 92% |
|  | Random effects model | 0.91 | 0.88,0.94 | < 0.001 | 92% |
| HbA1c (<7% vs. ≥7%) | Fixed effects model | 3.03 | 2.45,3.76 | <0.001 | 63% |
|  | Random effects model | 2.98 | 1.91,4.64 | <0.001 | 63% |
| HbA1c (<8% vs. ≥8%) | Fixed effects model | 2.25 | 1.50,3.38 | <0.001 | 0% |
|  | Random effects model | 2.25 | 1.50,3.38 | <0.001 | 0% |
| HOMA-IR | Fixed effects model | 1.00 | 0.97,1.02 | 0.75 | 83% |
|  | Random effects model | 0.91 | 0.72,1.14 | 0.400 | 83% |
| C-peptide level | Fixed effects model | 1.06 | 0.99,1.13 | 0.090 | 53% |
|  | Random effects model | 1.09 | 0.97,1.23 | 0.150 | 53% |
| Disease duration (per 1-year increase) | Fixed effects model | 0.89 | 0.88,0.90 | <0.001 | 43% |
|  | Random effects model | 0.89 | 0.88,0.91 | <0.001 | 43% |
| Disease duration (<5 vs. ≥5 years) | Fixed effects model | 3.41 | 2.35,4.96 | <0.001 | 48% |
|  | Random effects model | 3.84 | 2.15,6.86 | <0.001 | 48% |
| Number of oral antidiabetic agents | Fixed effects model | 0.63 | 0.54,0.74 | <0.001 | 70% |
|  | Random effects model | 0.56 | 0.38,0.81 | 0.002 | 70% |
| Insulin use | Fixed effects model | 0.31 | 0.28,0.35 | <0.001 | 91% |
|  | Random effects model | 0.34 | 0.23,0.52 | <0.001 | 91% |
| %TWL (per 1% increase） | Fixed effects model | 1.04 | 1.04,1.04 | <0.001 | 88% |
|  | Random effects model | 1.05 | 1.04,1.07 | <0.001 | 88% |
| %TWL (≥10% vs. <10%) | Fixed effects model | 2.98 | 2.66,3.35 | <0.001 | 38% |
|  | Random effects model | 2.99 | 2.57,3.48 | <0.001 | 38% |
|  |  |  |  |  |  |
|  |  |  |  |  |  |
|  |  |  |  |  |  |

**Supplementary Table 6.** Egger's Test Results for Publication Bias in the Included Studies

| Factors | P>\|t\| | 95%CI |
| --- | --- | --- |
| Age | 0.085 | -8.48,0.66 |
| BMI | 0.349 | -1.61,4.21 |
| HbA1c | 0.000 | -4.94,-2.07 |
| Disease duration | 0.990 | -1.26,1.24 |
| Insulin use | 0.812 | -3.17,3.95 |

**Supplementary Figure 1.** Sensitivity Analysis of the Included Studies.

**Supplementary Figure 2.** Funnel Plot for Publication Bias.
